# Real-Time Monitoring of a Sol–Gel Reaction for Polysilane Production Using Inline NIR Spectroscopy

**DOI:** 10.1021/acs.langmuir.3c00601

**Published:** 2023-05-28

**Authors:** Thomas Kisling, Robert Zimmerleiter, Lukas Roiser, Kristina Duswald, Markus Brandstetter, Christian Paulik, Klaus Bretterbauer

**Affiliations:** † Institute for Chemical Technology of Organic Materials, Johannes Kepler University Linz, Altenberger Straße 69, Linz 4040, Austria; ‡ RECENDT—Research Center for Non-Destructive Testing GmbH, Altenberger Straße 69, Linz 4040, Austria; § TIGER Coatings GmbH & Co KG, Negrellistraße 36, Wels 4600, Austria

## Abstract

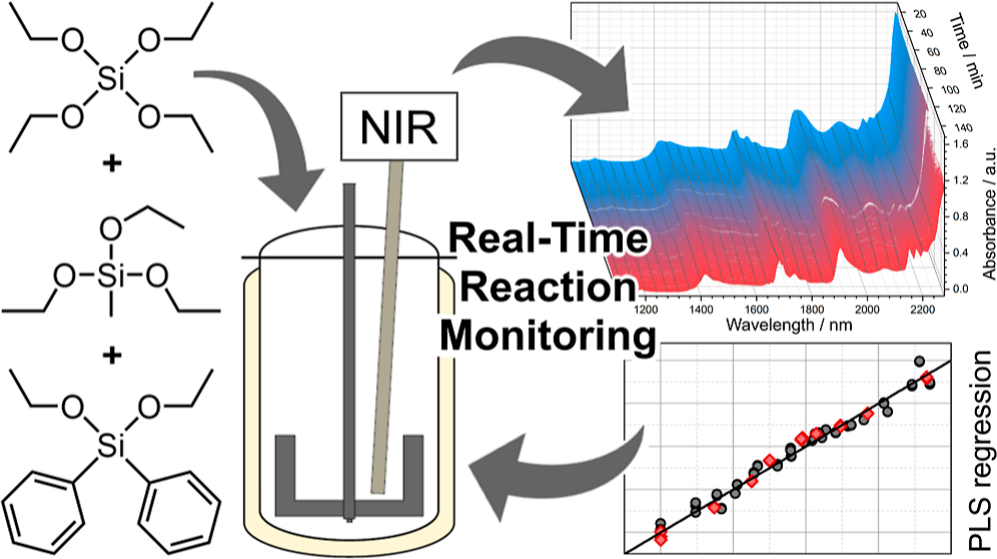

The sol–gel process is an effective method for the preparation of homogeneous structured nanomaterials whose physico-chemical properties strongly depend on the experimental conditions applied. The control of a three-component reaction with silanes showing multiple reaction sites revealed the need for an analytical tool that allows a rapid response to ongoing transformations in the reaction mixture. Herein, we describe the implementation of near-infrared (NIR) spectroscopy based on compact, mechanically robust, and cost-efficient micro-optomechanical system technology in the sol–gel process of three silanes with a total of nine reaction sites. The NIR-spectroscopically controlled reaction yields a long-time stable product with reproducible quality, fulfilling the demanding requirements for further use in coating processes. ^1^H nuclear magnetic resonance measurements are used as reference values for the calibration of a partial least squares (PLS) regression model. The precise prediction of the desired parameters from collected NIR spectroscopy data acquired during the sol–gel reaction proves the applicability of the calibrated PLS regression model. The determined shelf-life and further processing tests verify the high quality of the sol–gel and the produced highly cross-linked polysilane.

## Introduction

**Figure 1:**
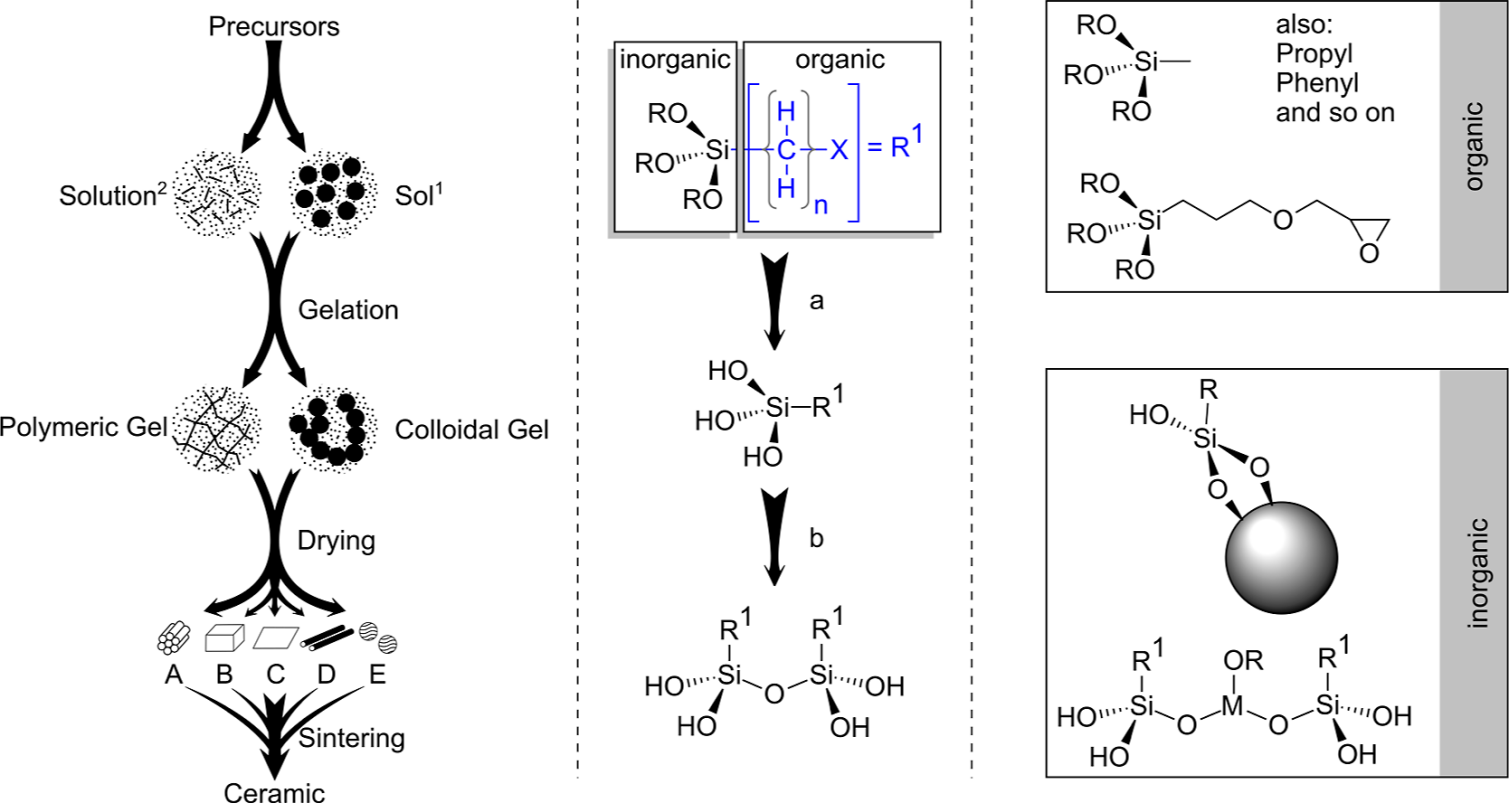
Schematic description of sol–gel reactions based on silane precursors. Left: transformation of the precursors into the sol or solution and adjacent gelation step resulting in a gel. Due to drying and sintering many different products can be achieved. Middle: The two main reactions hydrolysis (a) and condensation (b) are illustrated. R: mostly ethyl or methyl, X: various organic often functional groups containing moiety. Right: A selection of possible variations on the organic- or inorganic side of the precursors. The degree of silyl-crosslinking, functionalization, and resulting nanostructure enables a wide range of possible product properties.

Industrially applied coatings basically have two main functions: providing a decorative aspect and protecting the coated surface. For the decorative function, color, gloss levels, coating flow, and also surface textures play an important part.^1^ The economically most important task of coatings, however, is surface protection. Requirements vary greatly depending on the coating application of the final part and mostly include corrosion protection of the metal substrate, resistance against weather conditions (sunshine, rain, heat, and frost, combined with, e.g., ozone and salt fogs), and chemical resistance (not only acids, bases, solvents but also natural products such as tree resins or bird droppings).^1^ In addition to these common requirements, market demands for organic coatings include an increased temperature resistance as well as enhanced abrasion and scratch resistance. These demands are fulfilled by sol–gel materials that are also found in various high-tech applications ranging from optical materials,^2,3^ water-repellant textiles,^4,5^ to nanomaterials for medical applications.^6,7^ Sol–gel materials combine inorganic and organic structures forming a nano-scaled network obtained from the reaction of functionalized silanes. Tailor-made product properties are accomplished by the selection of suitable building blocks from a pool containing more than 3000 organic silanes available from standard chemical suppliers. Of this enormous monomer diversity, a wide range of different applications is feasible due to the high degree of flexibility in terms of tailored product properties. The sol–gel process describes the transition from a sol to a gel called gelation (Figure 1).^8,9^ The sol represents a stable liquid colloidal dispersion with particles smaller than 100 nm consisting of two or more components. The gel corresponds to a non-fluid colloidal or polymeric three-dimensional network whose voids are filled with a liquid medium.^10^ Depending on the fluid in the network, it refers to either a hydrogel or an alcogel (in the case of water or alcohol).^11^ The process condition for drying determines the obtained product microstructure whereby application of supercritical conditions results in an aerogel or when dried under ambient conditions a xerogel is formed in which the original gel structure is destroyed.^11^

Hydrolysis and condensation (Figure 1, middle) are not straightforward though because a large number of transformations play an important role in sol–gel reactions.^12^ In these complex processes, a wide variety of matter states, ranging from liquid to colloidal to solid, occur. At the beginning of the reaction, the precursors are hydrolyzed into their corresponding monomers. Depending on the number of alkoxy groups present in the monomer, this results in as many as four different product possibilities. In the reaction progress, condensation of these hydrolyzed species occurs, resulting in the sol. The range of variations is already enormous after one condensation step leading to the formation of different dimers and oligomers. Thus, with the ongoing condensation progress, the number of possible variations increases, making the process highly complex. Furthermore, hydrolysis and condensation cannot be completely separated from each other and occur in parallel during a long period of the reaction. The complexity is depending on the number of monomers and a prediction of the reactions taking place becomes increasingly difficult. However, especially during condensation, it is important to have an insight into the reaction progress. In the worst case, the required final product properties cannot be achieved, implying the necessity of a powerful analytical tool for reaction monitoring. Classical analyses in the field of sol–gel chemistry are usually done offline by sample extraction and often time-consuming measurement methods. Thus, no immediate real-time adjustment of the reaction conditions is possible, often resulting in out-of-specification product quality. The use of inline analytical methods results in increased reproducibility of the experiments and allows for predicting the required endpoint of the reaction to obtain a product that fulfills the specifications. Monitoring of critical reaction steps and prediction with real-time data and models based on the measurements allows a controlled reaction progress with exact endpoint determination.

Herein, for the production of a highly resistant coating, a sol–gel material and the associated production process are presented. A set of methods for inline- and offline-process analytics are described which allow the preparation of the polysilane with reproducible, constant product quality.

## Results and Discussion

**Figure 2:**
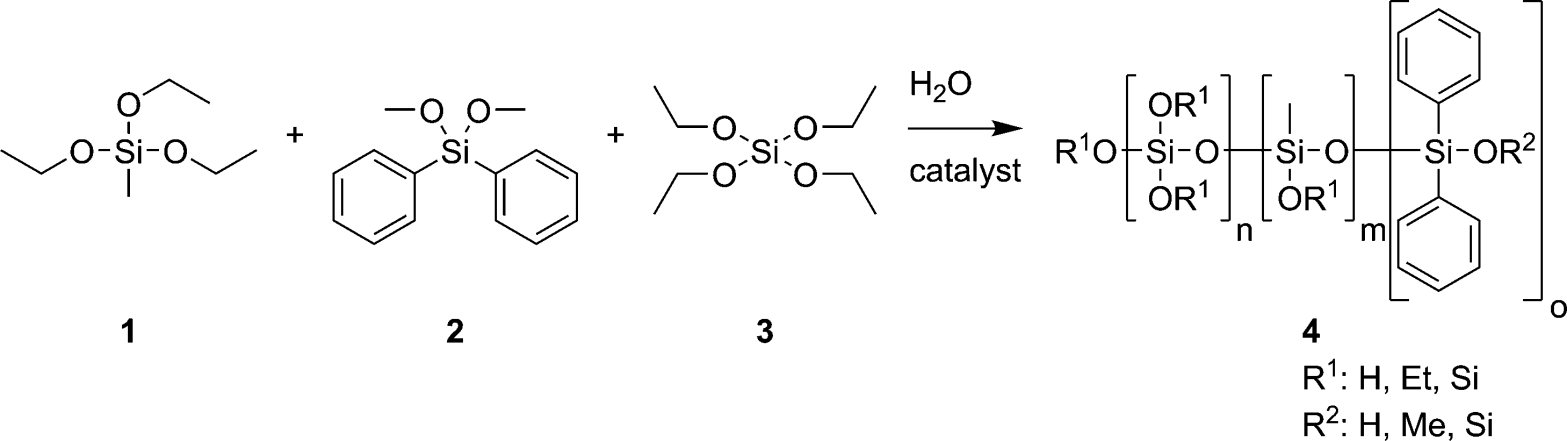
Reaction scheme for the sol–gel synthesis of the three-component polysilane **4** from MTEOS (**1**), DPDMS (**2**), and TEOS (**3**) with water and succinic acid as the catalyst. Coefficients n, m, and o are statistically distributed in the three-dimensional condensation network. Et: ethyl, Me: methyl.

From the pool of possible silane precursors available on the market, we screened 11 compounds according to their availability, cost-efficiency, their degree of methoxy or ethoxy functionalization, and the space requirement of the remaining moieties (methyl, phenyl, and cyclohexyl group). From the preliminary experiments, methyltriethoxy silane (**1**, MTEOS), diphenyldimethoxy silane (**2,** DPDMS), and tetraethoxy silane (**3,** TEOS) were selected due to their ability to form a flexible and simultaneously highly crosslinked network. Figure 2 shows the reaction scheme and silanes applied in the described sol–gel process as well as the obtained polysilane **4** processable in a coating formulation. TEOS (**3**) is used as the network former, while MTEOS (**1**) and DPDMS (**2**) are added to make the network more flexible.

**Figure 3:**
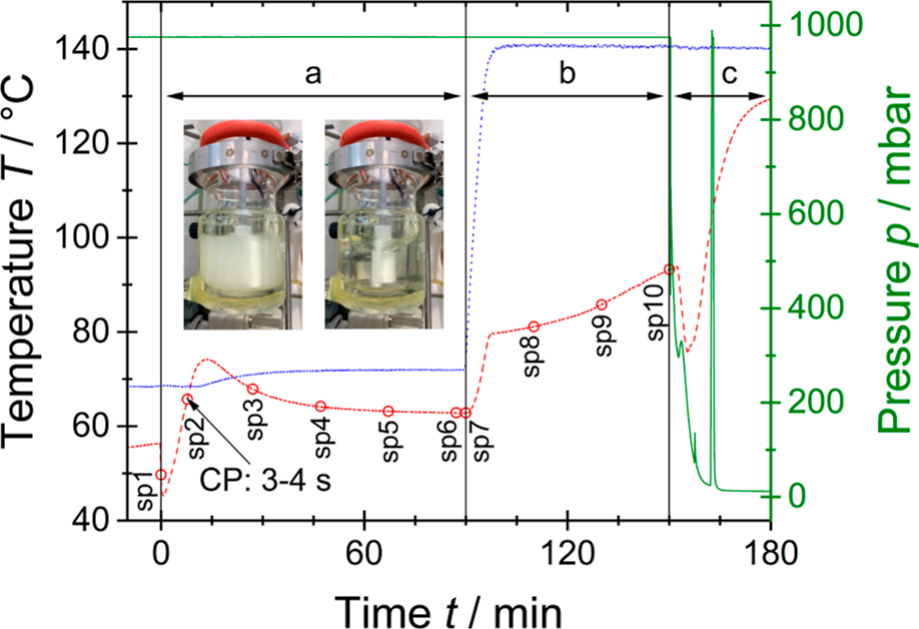
Progress of reaction temperature (red line, dashed), double-walled reactor mantle temperature (blue line, dotted), and pressure (green line, solid) over the reaction time. At the beginning of reaction phase (a), the temperature decreases due to the addition of the silane mixture. During the reaction phase (a), hydrolysis is favored, in the reaction phase (b), the temperature is increased, volatile reaction products are removed, and intense condensation occurs. Throughout the reaction phase (c), vacuum is applied for the complete removal of residual volatile compounds. The short loss of vacuum during reaction phase (c) is caused by a change from a waterjet vacuum to a high vacuum pump. sp1–sp10 mark sampling points for off-line measurement to determine the reaction progress. sp2 was directly taken after the clear point (CP) where the change of the reaction mixture from milky to clear lasts about 3–4 s. The inserted pictures show the transformation of the reaction mixture at the CP.

For synthesis, a double-wall glass reactor heated with an external heating circuit, equipped with a mechanically driven anchor stirrer, a reflux condenser, and a water separator was used (Figure S1, Supporting Information). The reaction was monitored with a polytetrafluoroethylene (PTFE) encased thermocouple, a pressure sensor as well as an inline NIR transflection measuring probe connected via optical fibers to a compact micro-optoelectromechanical system (MOEMS)-based NIR-spectrometer. Figure 3 shows the reaction phases, related conditions, and sampling times for a typical synthesis batch. An equimolar amount of water (calculated on hydrolyzable groups) and succinic acid as the catalyst are fed into the reactor and heated to 60 °C. After a stable temperature is reached, the silane mixture **1–3** is added while stirring vigorously. Immediately upon addition, the mixture turns milky and the exothermic hydrolysis reaction starts. In the case of succinic acid as the catalyst, the so-called clear point (CP) is reached after approximately eight minutes when the milky reaction mixture forms a clear, colorless solution (Figure 3, inserted pictures). Stronger Brønsted acids accelerate the reaction, and thus, the clear point is reached much faster. For succinic acid as the catalyst, the exothermic reaction causes an increase of the reaction temperature up to typically 75 °C. Thereafter, the temperature steadily flattens to 60 °C. The reaction phase (a) is kept for 90 min and subsequently, the temperature of the heating jacket is set to 140 °C for 60 min to remove most of the volatile reaction products ethanol, methanol, and water and simultaneously favor condensation (Figure 3, reaction phase b). Finally, a vacuum is applied at 140 °C to remove residual volatile components from the reaction mass (Figure 3, reaction phase c), yielding polysilane **4** as a highly viscous, slightly yellow melt. During the experiment, the double-wall reactor mantle temperature, the reaction temperature, and the pressure (quality of the vacuum in reaction phase c should be below 10 mbar) were recorded since the adherence to the temperature, pressure, and time course is of great importance for a successful and reproducible sol–gel reaction as well as for achieving the desired product properties.

First syntheses without reaction control yielded products with strongly varying properties ranging from solid and easily processable as desired to sticky or completely hardened materials without the possibility for further processing. Especially for upscaling, it became apparent that the synthesis must be carefully monitored, and with the implementation of time-resolved sampling and off-line analyses, the reaction can be followed to ensure a stable process and to determine the optimum endpoint. Sticky products show an insufficient degree of condensation, are difficult to grind, and were not stable enough upon storage. In contrast, excessive condensation yields products that influence the processing in coating formulations, e.g., melt flow properties and hindered layer formation (blistering, crack-formation, or flaking), or can even destroy the synthesis equipment by completely curing inside the reactor. Thus, detailed reaction monitoring turned out to be essential for a predictable and reproducible reaction progress. Analytical samples were directly taken from the reaction mass–the reaction was not stopped for sampling, immediately cooled to room temperature, and forwarded to attenuated total reflection mid-infrared spectroscopy (ATR-MIR) and ^1^H nuclear magnetic resonance (NMR) analyses. A consistent sampling procedure concerning the temporal sequence appeared as a crucial factor for reproducible analytical results.

**Figure 4:**
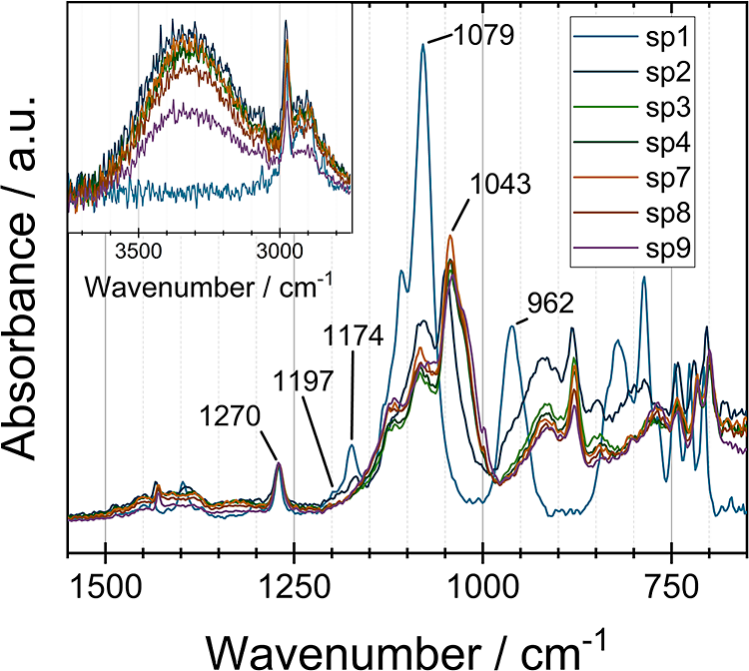
ATR-MIR spectra of the samples sp1–4 and sp7–10 were taken during the reaction. sp5 and sp6 are not included for reasons of clarity. The spectra were normalized to the methyl signal at 1270 cm^–1^. Signals at 962, 1079, and 1174 cm^–1^ are related to Si-OC_2_H_5_ (stretching), 1043 cm^–1^ from Si–O–Si (stretching), 1197 cm^–1^ and 1079 cm^–1^ from Si–OCH_3_ (stretching), and 1270 cm^–1^ from Si–CH_3_ (deformation). The disappearing of Si-OC_2_H_5_ and Si–OCH_3_ related signals and the appearance of Si–O–Si stretching band indicates hydrolysis and condensation reactions.

ATR-MIR is a well-established and fast method to obtain insight into the reaction progress by revealing significant changes in the reaction mass. Figure 4 shows the results of ATR-MIR measurements during the sol–gel reaction. For better comparability, the acquired spectra were normalized to the Si–CH_3_ signal of MTEOS (**1**) at 1270 cm^–1^^13^ since this signal is clearly assignable in all spectra (sp1–sp10). The reduction of the signal at 1079 cm^–1^ shows the proceeding hydrolysis of the alkoxy groups, as this absorption band can be attributed to Si-OC_2_H_5_ as well as Si–OCH_3_ groups.^13^ Inversely, a signal at 1043 cm^–1^ appears from sp2 onward, which is caused by the formation of Si–O–Si bonds and thus becomes increasingly larger as condensation proceeds.^13^ Therefore, it is possible to assign those characteristic signals to the hydrolysis and condensation reactions. Especially due to the overlapping of individual signals, small changes are difficult to detect and quantitative statements about the processes in the reaction mass are limited. Thus, it was not possible to calculate the amount of free water in the system based on the signal at 3000–3600 cm^–1^ since the OH stretching vibration of ethanol and methanol is also located in this spectral range.

**Figure 5:**
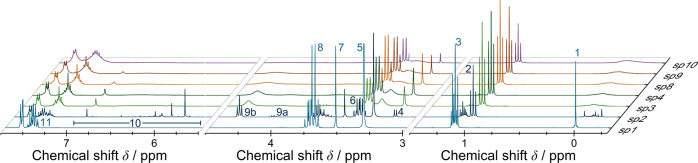
^1^H NMR spectra of the samples taken during the reaction (sp1–sp4 and sp8–sp10). Due to only minor changes from sp5 to sp7, these points were not included. The starting silane mixture **1**–**3** is shown in track sp1. Disappearing of signals annotated with 3, 7, and 8 (−OCH_3_ and OCH_2_CH_3_) and arising of 2, 4, 6, and 9–10 (methanol, ethanol, and silanol-OH) proves hydrolysis. From sp2 onward, condensation is indicated via the disappearance of silanol-OH signals 10 in the range of 5.5–7 ppm.

To obtain quantitative information about the sample composition, ^1^H NMR measurements were applied in the next step. Figure 5 shows the change of chemical composition during hydrolysis and condensation by means of the ^1^H NMR spectrum at sp1–sp4 and sp8–sp10.

Signal areas were referenced to the separated methyl-signal at 0 ppm (Figure 5, sp1, signal assigned with 1). The broadening of the methyl signal is attributed to the changing chemical and magnetic environment during the reaction. Signal 3 in sp1 at 1.16 ppm is assigned to the ethoxy–CH_3_–groups of MTEOS (**1**) and TEOS (**3**) that is already significantly reduced due to hydrolysis at sp2 after 8 min reaction time. In turn, a triplet assigned as signal 2 at 1.06 ppm appears from sp2 onward (Figure 5, sp2–sp4 and sp8–sp10), which originates from the CH_3_ group of ethanol formed by hydrolysis. The signal increases until sp8 and thereafter decreases in sp9 and sp10 due to the evaporation of volatile reaction products in reaction phase b (Figure 3). Furthermore, quartet 6 originates from the methylene group in ethanol showing a similar intensity change discussed for signal 2 (ethanol–CH_3_–group). Signal 4 at 3.18 ppm, assigned to the methyl group of the hydrolysis product methanol, appears in sp2 and increases until sp8 and subsequently decrease due to evaporation in reaction phase b (Figure 3). Signal 5 at 3.33 ppm is caused by the water in the reaction mass. Reduction of signal intensity indicates consumption of water during the hydrolysis of silanes **1–3**. Although reactants were used equimolar, the water signal never disappears indicating a simultaneous hydrolysis and condensation reaction that concurrently consumes and releases water. Signal 8 (Figure 5) at 3.76 ppm, represents the CH_2_ group of the ethoxy moiety of silanes **1** and **3** that behaves in the opposite direction to signals 6 and 2 and disappears completely in sp3 (Figure 5). Singlet 7 at 3.56 ppm is assigned to the CH_3_ group of the methoxy functionality in DPDMS (**2**). Signals 9a and 9b at 4.10 ppm and 4.36 ppm originate from the OH signal of the formed methanol and ethanol. Region 10 in the range of 5.6–7.0 ppm (Figure 5) summarizes OH signals of the hydrolysis products of silanes **1**–**3**. In the first eight minutes of the reaction, distinct OH signals are observed (sp2, Figure 5) that coincide with a singlet at 6.92 ppm (sp 3–9) during the progressing hydrolysis and condensation. Within sp9 these signals nearly completely disappear showing a high degree of condensation. The aromatic protons of silane **2** are allocated in the area of 7.25–7.68 ppm (signal assignment 11, Figure 5). Broadening of the signals indicates a successful uptake in the network of the sol–gel product **4**.

Time-resolved ^1^H NMR measurement enables qualitative and quantitative information about hydrolysis and condensation (appearance and disappearance of compound characteristic signals and signal integrals, respectively). However, these results still represent only snapshots, which are measured offline and thus a time delay of a few minutes is unavoidable, which can lead to out-of-specification products. To obtain a material that is further processable for coatings, a successful sol–gel reaction must be stopped at a high degree of hydrolysis and condensation in the range of 95–97% determined by stability measurements of aged products. The resulting low number of reactive functionalities (silanol-OH, ethoxy-, and methoxy-groups) of 3–5% ensures storage stability over several months at temperatures between 4 and 30 °C.

As the method of choice for inline monitoring of the analyte concentrations in the reactor, NIR spectroscopy was chosen since it is a highly promising measurement method to directly deliver real-time process information. NIR-spectroscopy, usually combined with multivariate data analysis methods, has been utilized for many decades for this purpose in various industrial sectors including the pharmaceutical,^14,15^ chemical,^16,17^ and food industry^18^ as well as for medical applications.^19^ The widespread applications of NIR spectroscopy for process monitoring are routed in the advantageous properties of this measurement technique such as its non-destructive nature, inline-capability, easy maintenance, the possibility for the simultaneous determination of multiple target analytes, and short measurement times. With the increase in the availability of cost-efficient and compact spectrometers based on MOEMS technology in recent years, hardware costs for NIR sensing applications have been significantly lowered, making this technology an even more attractive option. The potential of MOEMS-based NIR spectrometer technology has already been successfully demonstrated in various spectroscopic applications in both science and industry.^20−22^ Herein, we utilize this technology for the real-time monitoring of the conducted hydrolysis and condensation batch reaction. Spectral data were collected with a MOEMS-based broad-band Fourier-transform near-infrared (FTNIR) spectrometer connected to a transflection measurement probe in the range from 1100 to 2500 nm. A schematic drawing of the NIR-measurement setup shows Figure S2 (Supporting Information) and a photograph of the reactor with inserted NIR-transflection probe is provided in Figure S1. The acquired absorbance spectra were preprocessed using Savitzky–Golay filtering, multiplicative scatter correction (MSC), and mean centering. Due to the low light intensity above 2280 nm caused by low light emission of the used halogen lamp as well as strong absorption in the optical fibers and process medium, only wavelengths between 1100 and 2280 nm were considered for the analysis.

**Figure 6:**
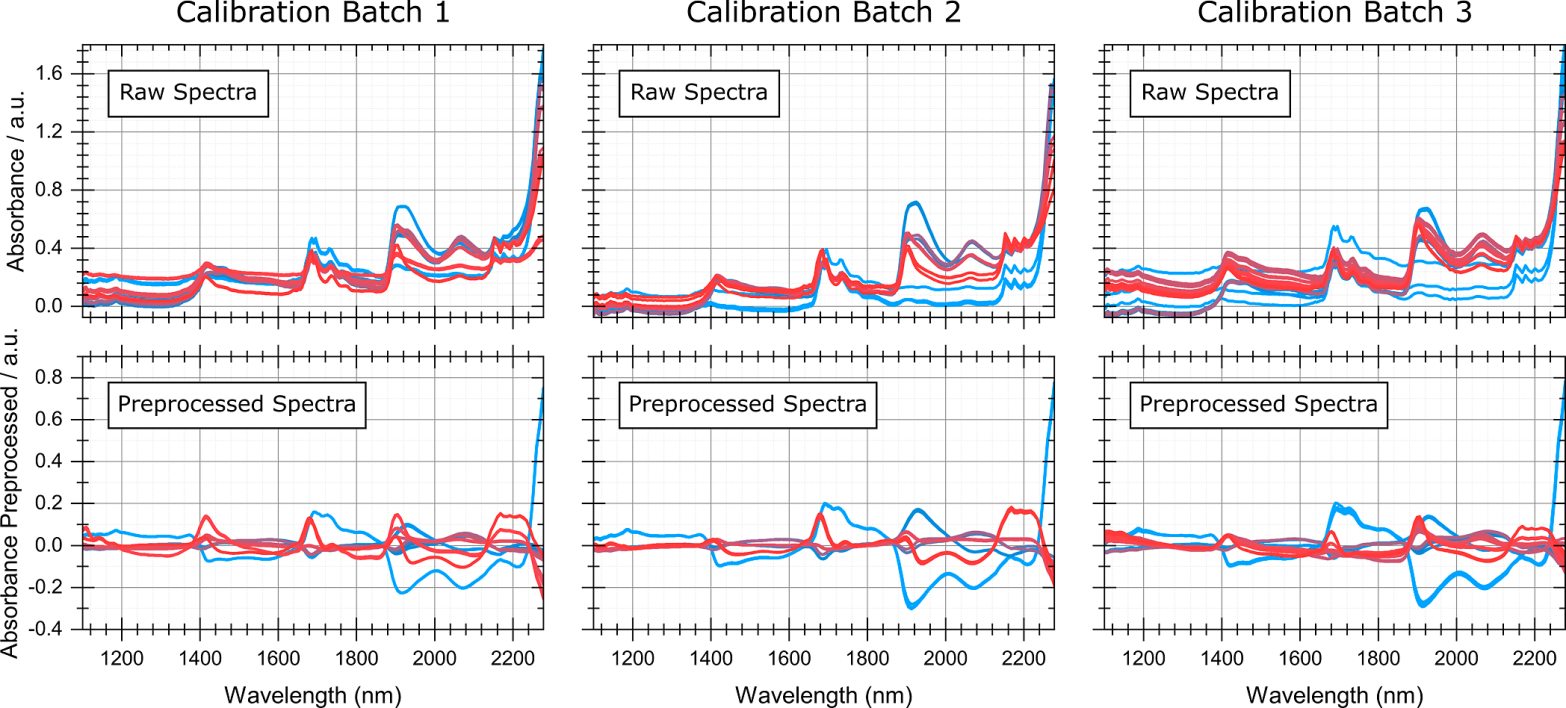
Spectra used for calibration of the PLS-model. The top row shows the raw absorption spectra and the bottom row the same spectra after preprocessing, clearly showing the strong reduction of unwanted disturbances in the spectral data. Each spectrum is color-coded from red to blue for the process time for the respective batch. Three spectra were used for each sampling point (plotted in the same color). A total of 12 reference measurements were made for calibration batches 1 and 3, while only 6 were made for calibration batch 2 for operational reasons.

A single partial least squares (PLS) regression model^23^ with eight latent variables (LVs) was calibrated using the preprocessed inline absorbance spectra with the matching offline data for six investigated analytes (water, ethanol, methanol, silanol groups, ethoxy groups, and methoxy groups) obtained via integration of the analyte-specific signals in the acquired NMR spectra for three calibration batches. The spectra used for the calculation of the PLS regression model are shown in Figure 6, both as raw absorbance spectra and after the application of the aforementioned preprocessing methods. Therein, it can be clearly seen that most of the unwanted disturbances in the spectral data are effectively removed by the application of spectral preprocessing. The number of LVs was chosen via minimization of the cross-validation error using the *Venetian blinds* method with a blind thickness of three (since three subsequently recorded spectra were used per reference point) and 10 data splits. A fourth independent batch process was carried out for validation of the calculated PLS-regression model to assess its performance. Figure S3 (Supporting Information) shows the acquired raw spectra for the validation batch as a function of time.

**Figure 7:**
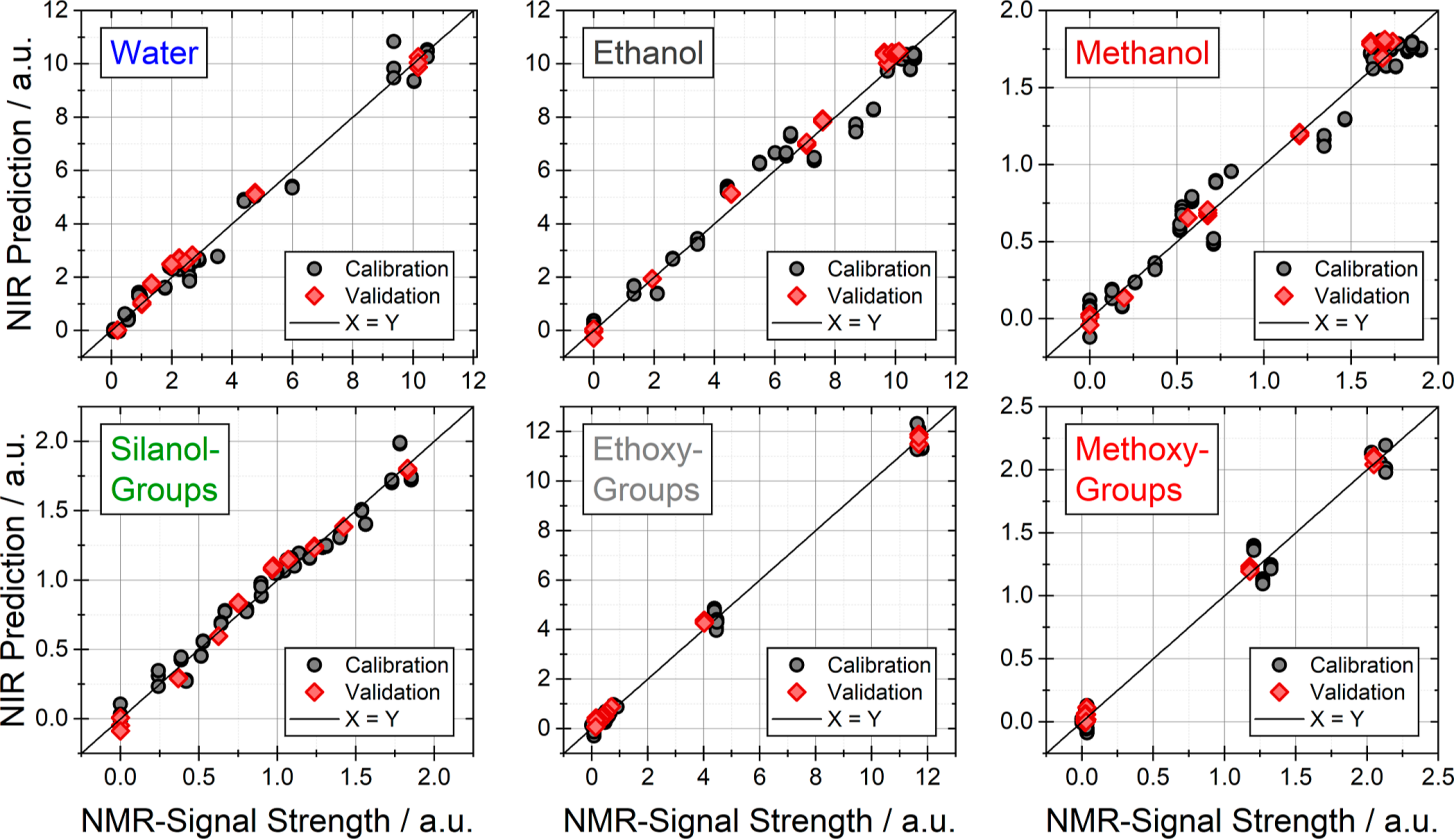
Values for the six analytes were calculated via the measured inline NIR-spectra via PLS-regression plotted against NMR-signal strength which is given by the integral over the substance-specific peaks in the NMR-spectrum. for the three calibration batches (gray) and the validation batch (red). As a reference, the ideal 1:1 curve is shown as a black line.

Figure 7 shows the values predicted from the acquired NIR absorption spectra plotted versus the offline reference measurements using NMR for the six analytes. Good agreement was achieved for all six analytes with *R*^2^ values > 0.98 for the validation batch and >0.97 for the three calibration batches. Results of the PLS-regression are summarized in Table 1.

**Table 1:** Results of PLS Regression for Calibration and Validation Batches

analyte	RMSEC^a^/a.u	NRMSEC^b^/a.u	*R*^2^ (cal)	RMSEP^c^/a.u	NRMSEP^d^/a.u	*R*^2^ (val)
water	0.382	0.0365	0.979	0.336	0.0321	0.992
ethanol	0.514	0.0483	0.981	0.403	0.0379	0.996
methanol	0.114	0.0600	0.973	0.085	0.0448	0.994
silanol-groups	0.079	0.0426	0.997	0.074	0.0399	0.981
ethoxy-groups	0.209	0.0177	0.996	0.153	0.0130	0.999
methoxy-groups	0.069	0.0324	0.990	0.037	0.0174	0.998

a Root mean square error of calibration (RMSEC).

b RMSEC normalized by division with range of measured concentrations of the respective analyte.

c Root mean square error of prediction (RMSEP).

d RMSEP normalized by division with range of measured concentrations of the respective analyte.

**Figure 8:**
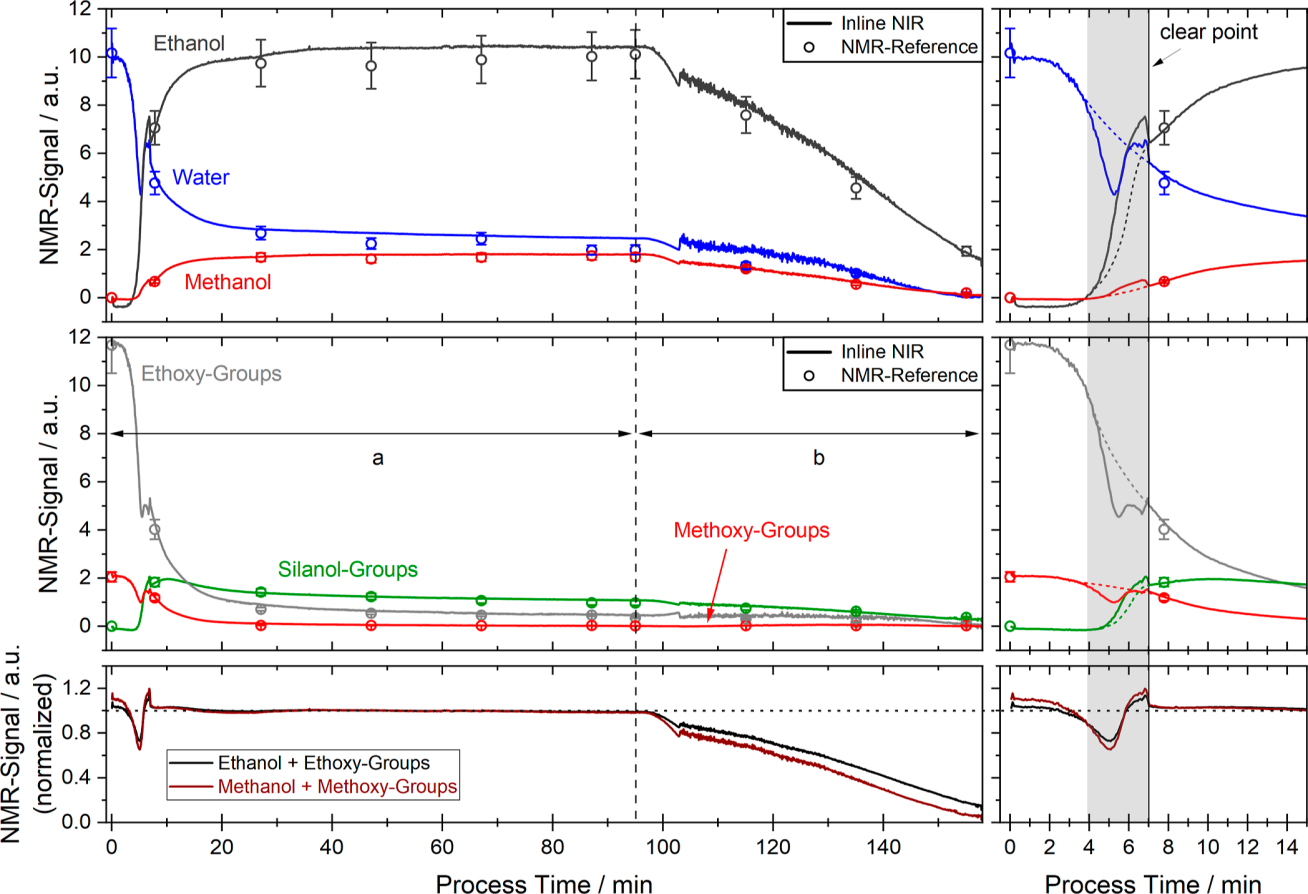
Values calculated from the inline NIR-spectra plotted against the process time of the validation batch for the six analytes. Offline reference values obtained by NMR are shown as circles with indicated error bars. The timeframes indicated by “a” and “b” correlate to reaction phase (a) and reaction phase (b), as indicated in Figure 3. The bottom graph shows the normalized sum of ethanol plus ethoxy-groups and methanol plus methoxy-groups for the same timeframe. Zoomed-in graphs for the first 15 min are shown on the right side.

Concentrations of the analytes plotted versus time for the validation batch are presented in Figure 8. Therein, also the offline NMR measurements are indicated as circles at the respective sampling time which correlate very nicely with the real-time values calculated from the NIR-spectra using the PLS regression model. Offline NMR measurements are drawn with an estimated error bar of 10% of the measured value. Since the strongest changes in the analyte concentrations happen at the beginning, a closer look at the first 15 min of the process is shown in the zoomed-in graph on the right side.

The real-time signals calculated from the NIR-spectra reveal, that after the addition of silanes for the first 2–3 min the signals for all analytes are nearly constant as the temperature in the reactor increases. After a high enough temperature is reached, the exothermic hydrolysis reaction accelerates and further increases the temperature in the reactor. It is observed that both the water concentration as well as the number of ethoxy-groups rapidly decreases until both reach a minimum at about 5.5 min. A very similar trend as for the ethoxy-groups can be seen for the methoxy-groups, albeit on a smaller signal amplitude. In the same timeframe, the signals for ethanol and methanol increase as they are formed from the ethoxy- and methoxy-groups, respectively, while consuming the water in the process medium in the occurring hydrolysis reaction.

The sum of the signal intensities of ethoxy-groups and ethanol as well as methoxy-groups and methanol (normalized to their respective value at 60 min) are shown in the bottom graph in Figure 8. It is expected that these added signals remain constant in reaction phase (a) since no material is added or removed from the batch reactor. Significant deviations are observed in the timeframe of 4–7 min, indicated as the gray-shaded area in Figure 8 on the right. These deviations can be mainly attributed to the strong changes in the optical density of the process medium in this timeframe. With the increase in concentrations of both ethanol and methanol as well as temperature, the solubility for the silanes in the emulsion increases, which leads to a decreased average droplet size in the process medium (change from cloudy two-phase mixture to clear solution). Since the scattering coefficient is strongly dependent on the average particle size and the wavelength, this influences the measured NIR-absorption spectrum. Consequently, this can lead to artifacts in the concentrations calculated from the spectra using PLS-regression, especially if different optical densities of the medium were not included in the model calibration. Regarding this inconsistency, the predicted values in the timeframe of 4–7 min for the six investigated analytes most likely cannot be trusted. This assumption is supported by the temporal progression of the concentrations. The signals here exhibit a sharp decrease at the clear point and/or zigzagging, which are not explainable from a chemical point of view. The much more likely progression of the signal intensities in this timeframe was roughly estimated and indicated as dashed lines in the respective color.

After the clear point at approximately seven min is reached, both normalized added signal intensities jump back to the expected value of 1 and remain there until the end of reaction phase (a) as expected. At the same time, the predicted concentrations for the six analytes return to chemically reasonable trajectories.

With the start of reaction phase (b), as the temperature in the reactor increases, the volatile components water, ethanol, and methanol evaporate, apparent in their respective signals. The normalized signals in the bottom of Figure 8 show that the decrease in the methanol-signal happens slightly earlier and steeper as for ethanol, due to the lower boiling point of methanol (64.7 °C) compared to ethanol (78.2 °C). As the temperature in the reactor further increases, the process medium starts boiling heavily. Due to bubbles forming inside the measurement slit of the NIR transflection probe and generally much more turbulence in the process medium, the noise on the measured NIR-spectra increases which also appears as increased noise in the predicted concentration of all analytes at around 103 min. Despite this increased noise, the predictions of the PLS-regression model give values very close to the offline reference measurements and nicely resemble the expected trajectory of the analyte concentrations in the reactor.

**Figure 9:**
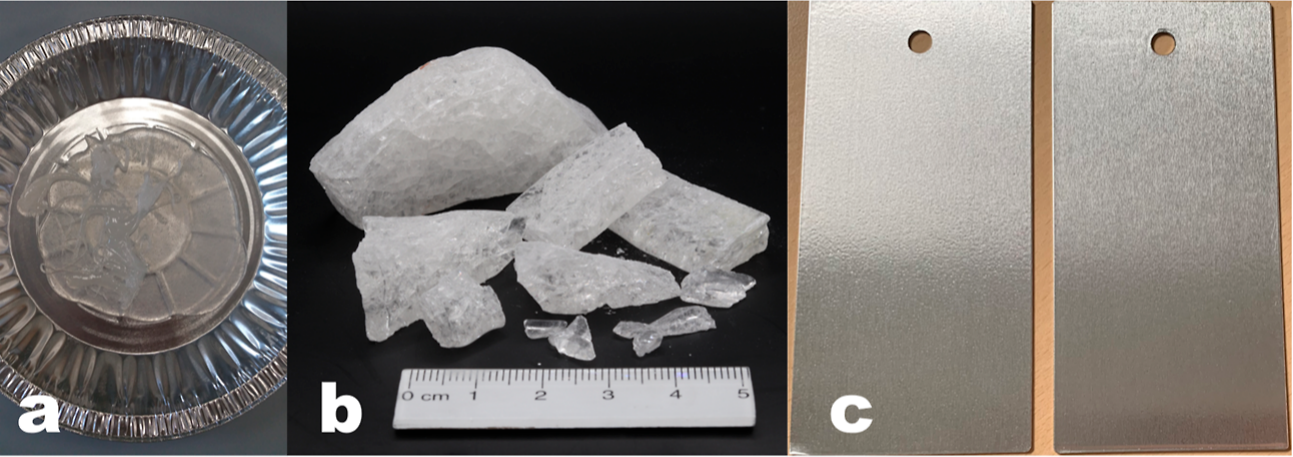
Polysilane **4** obtained from the inline NIR spectroscopy monitored three-component sol–gel reaction. (a) The synthesis affords **4** as a hot melt that is drained into an aluminum tray (300 mm diameter) to cool to room temperature. (b) The transparent and brittle polysilane plate is broken into rough pieces for storage. (c) Aluminum plates coated with polysilane **4** (50 × 100 mm). The coating on the left plate shows slight blistering, while the coating on the right plate shows no cracks, blistering, or flaking and therefore passes the demands for the visual check.

Polysilane **4** is obtained from the synthesis as a hot melt that was drained from the reactor into an aluminum tray to cool to room temperature, yielding **4** as a transparent and brittle plate (Figure 9a). Grindability, re-meltability, and curability are important parameters for the applicability of coating formulations. Thus, **4** should not stick together or harden during storage. Polysilane **4** broken into rough pieces (Figure 9b) shows storage stability at room temperature and under the exclusion of moisture for several months. For application-oriented tests, **4** was finely ground with a lab mill and the powder was distributed homogeneously on an aluminum sheet. Polysilane **4** is then melted at 120 °C in a heated cabinet and the metal sheet is coated with a two mils doctor blade, affording a 50 μm thick coating. Subsequently, **4** is cured at 200 °C for 30 min (Figure 9c). The hardening process, described by crosslinking and exclusion of volatile reaction products (water, ethanol, and methanol), causes a significant shrinkage resulting in a 20–25 μm thick transparent coating.

Storage stability and processability are directly related to the degree of condensation and the content of free silanol-, methoxy-, and ethoxy-groups that were inline monitored during the formation of **4** with the inline NIR measurements. Polysilane **4** with a degree of condensation between 95 and 97% showed the best storage stability and processability. Materials with a degree of condensation outside that window showed blistering (Figure 9c, left plate), crack formation, and in the worst case flaking during the curing process. The visually approved coatings are highly smooth and showed no cracks, blistering, or flaking of the layer (Figure 9c, right plate). Furthermore, a cross-cut adhesion test was used to evaluate the adhesion of the coating to the aluminum sheet. The best results were obtained for highly smooth coatings estimated with degree 1–2 (small flakes of the coating have detached affecting not more than 15% of the cross-cut area). The cross-cut test was performed and evaluated according to the standard for paints and varnishes (ISO 2409:2013) that distinguishes into six categories (0: best result with no detachment of edges—5: more than 65% of the cross-cut area is detached). The above-mentioned results for storage stability, processability, and surface properties represent polysilane **4** batches synthesized with an optimized ratio of starting materials and NIR-monitored reaction conditions.

## Conclusions

For the three-component sol–gel reaction between MTEOS (**1**), DPDMS (**2**), and TEOS (**3**), a NIR measurement-based inline reaction control is implemented to overcome a limited reaction monitoring from offline ATR-MIR and ^1^H NMR measurements. The classical offline ATR-MIR and ^1^H NMR measurements provide the underlying calibration set for the partial least squares regression model that allows monitoring of the main components in real-time, which can further be used to identify deviations from the expected process trajectory and tightly control the process conditions for optimal quality of the final product. Within the NIR inline reaction control, the six main analytes (starting materials and intermediates of the partial reactions) are monitored and evaluated simultaneously, which enables an accurate endpoint determination for the condensation step in the multicomponent sol–gel reaction. Thus, the NIR inline reaction control using a MOEMS-based FTNIR-spectrometer constitutes a cost-efficient and reliable method to assure reproducible process control. Polysilane **4** could be synthesized in reproducible quality with storage stability at room temperature over several months. The synthesized products were applicable in the production of coatings showing no blistering, cracks, or flaking of the layer. The obtained temperature-hardened, highly smooth coatings with about 25 μm thickness were estimated class 1–2 in the cross-cut test according to standard ISO 2409:2013.

## Experimental Section

All substances used were of technical or higher quality. Reagents were purchased from standard chemical suppliers and used without further purification. For ^1^H NMR measurements, a Bruker Avance III/300 (300 MHz) spectrometer with standard pulse sequences as provided by the manufacturer was used. Analytical samples were directly taken from the reaction. The reaction was not stopped for sampling. Samples were taken with a pipette. 12 μL of reaction medium were dissolved in 0.6 ml of deuterated dimethylsulfoxide (DMSO-*d*_6_, 99.8%) before the measurement and spectra are referenced on the solvent signal at 2.50 ppm. NIR spectra were recorded with a transflection measurement probe (Solvias AG, Switzerland) with a total optical path inside the medium of 2 mm. The probe was connected to a halogen light source (Avantes BV, Netherlands) and a MOEMS-based broad-band Fourier-transform near-infrared (FTNIR) spectrometer (Hamamatsu, Japan) via suitable optical fibers. Spectral data were recorded approximately every 4 s in the wavelength range from 1100 to 2500 nm using the spectrum of air taken prior to the start of the batch process as background for absorbance calculation. For ATR-MIR measurements, a Nicolet 5700 ATR-IR device was used.

General procedure: a double-walled reactor is equipped with a mechanical anchor stirrer, a reflux condenser, a PTFE-encased thermocouple K, and a water separator. The system is heated with a Julabo MV-4 as an external heating circuit. Succinic acid dissolved in water is brought to the reaction temperature of 60 °C and the mixture of MTEOS (1), DPDMS (2), and TEOS (3) is added while stirring vigorously. The reaction mixture is stirred at 60 °C for 90 min and then heated to 150 °C for 60 min. Subsequently, a vacuum is applied to remove the volatile compounds until the desired degree of condensation is reached. The hot reaction mixture is poured into aluminum ladles for cooling, and polysilane 4 is obtained as a white powder after grinding in a lab mill.

## Supporting Information Available

Photograph of the used double-wall glass reactor, schematic drawing of the used NIR-measurement setup, and raw absorbance spectra acquired for the validation batch (PDF)

## Abbreviations

NIR: near-infrared
MTEOS: methyltriethoxy silane (**1**)
DPDMS: diphenyldimethoxy silane (**2**)
TEOS: tetraethoxy silane (**3**)
PTFE: polytetrafluoroethylene
CP: clear point
ATR-MIR: attenuated total reflection mid-infrared
NMR: nuclear magnetic resonance
MOEMS: micro-optoelectromechanical system
FTNIR: Fourier-transform near-infrared
MSC: multiplicative scatter correction
PLS: partial least squares
LV: latent variable
RMSEC: root mean square error of calibration
NRMSEC: normalized root mean square error of calibration
RMSEP: root mean square error of prediction
NRMSEP: normalized root mean square error of prediction.
