# Supplementary material for: Real-Time Monitoring of a Sol–Gel Reaction for Polysilane Production Using Inline NIR Spectroscopy
Source: Langmuir. 2023 May 28;39(23):8153–62. doi: 10.1021/acs.langmuir.3c00601 (PMC10269431; doi:10.1021/acs.langmuir.3c00601)
Supplement: Supplementary file 1 — la3c00601_si_001.pdf [file la3c00601_si_001.pdf]

Supporting Information

Real-Time Monitoring of a Sol-Gel Reaction for Polysilane Production Using Inline NIR Spectroscopy

Thomas Kisling[a], Robert Zimmerleiter[b], Lukas Roiser[c], Kristina Duswald[b], Markus Brandstetter[b], Christian Paulik[a], and Klaus Bretterbauer[a]*

[a] Institute for Chemical Technology of Organic Materials, Johannes Kepler University Linz, Altenberger Straße 69, 4040 Linz, Austria

[b] RECENDT – Research center for Non-Destructive Testing GmbH, Altenberger Straße 69, 4040 Linz, Austria

[c] TIGER Coatings GmbH & Co KG, Negrellistraße 36, 4600 Wels, Austria

[*] Corresponding Author - Email: klaus.bretterbauer@jku.at

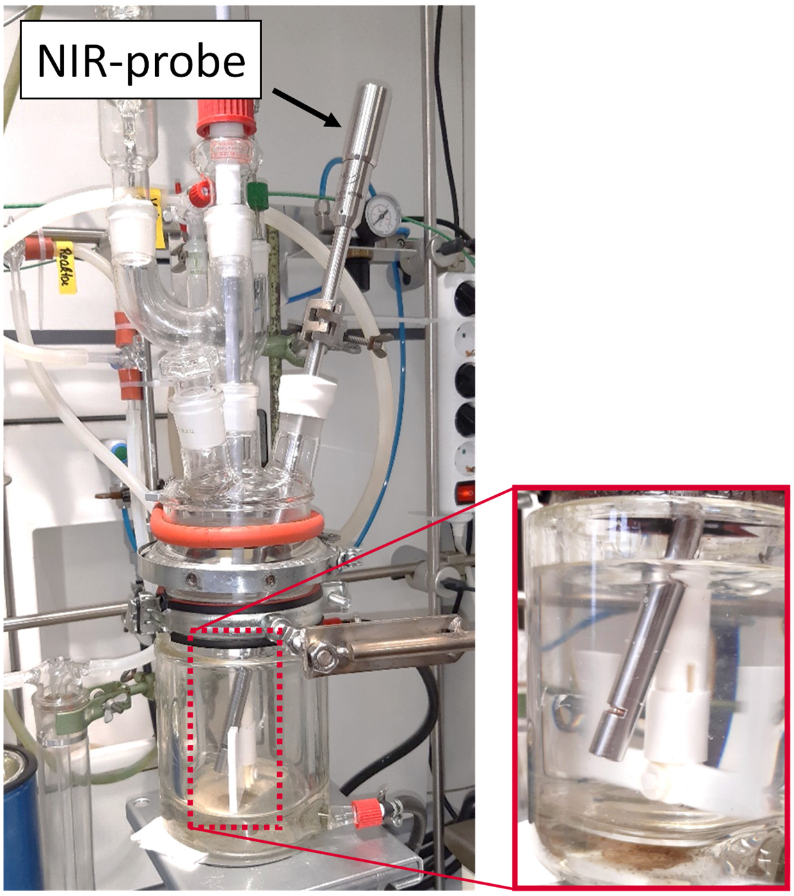

Figure S1. Photograph of the used double-wall glass reactor with inserted NIR-Probe. The zoomed area (right picture) shows a closeup of the probe tip and the measurement slit.

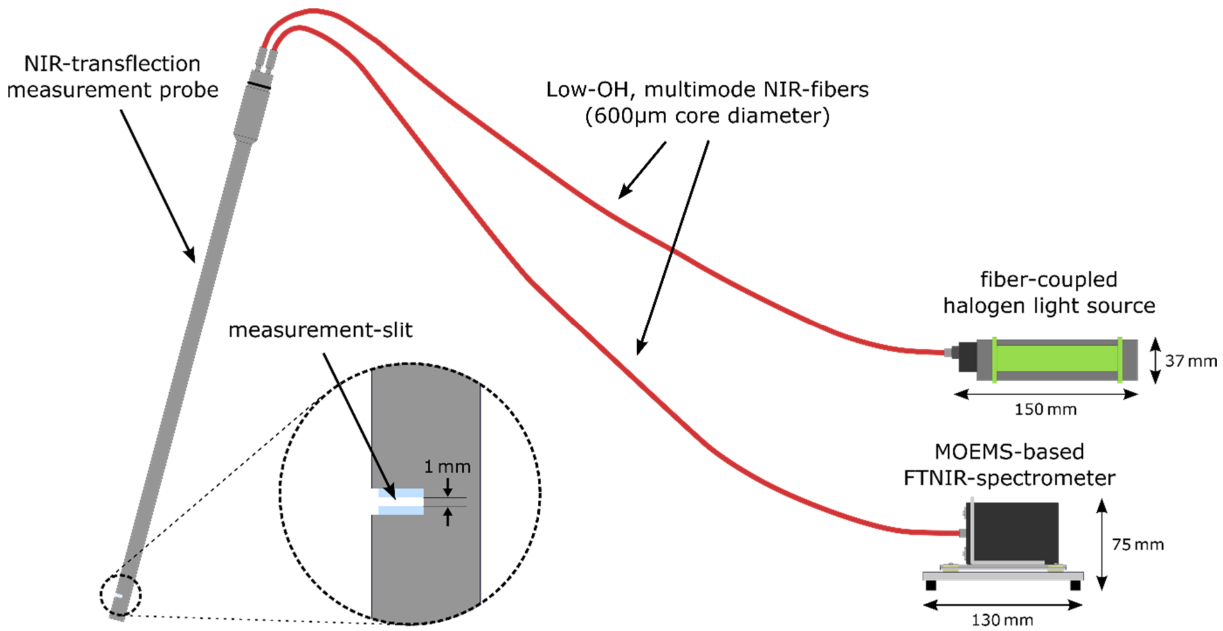

Figure S2. Schematic drawing of the used NIR-measurement setup. A NIR-transflection probe was connected via two multimode NIR-Fibers to a fiber coupled halogen light source and a compact MOEMS-based FTNIR-spectrometer. Chemical information is acquired inside the measurement slit, where the light propagates twice through the process medium.

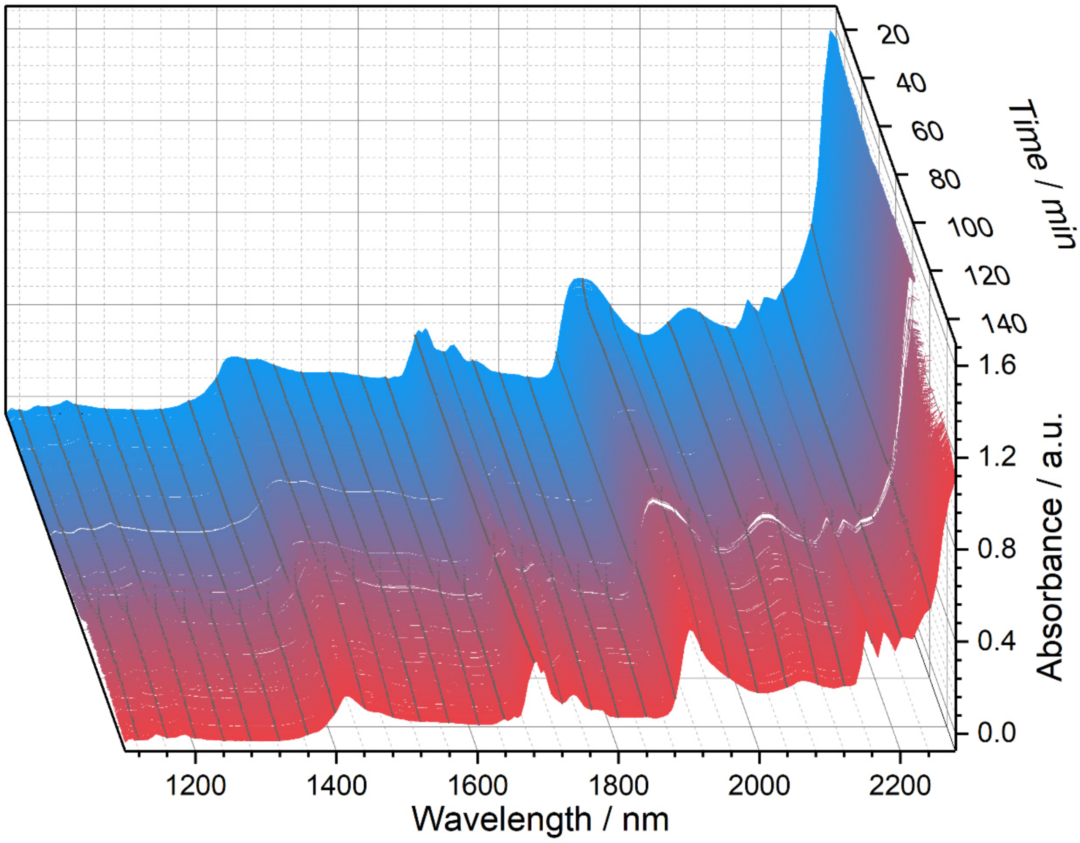

Figure S3. Raw absorbance spectra acquired for the validation batch; process time is color-coded from red to blue. Absorbance values at 40 nm intervals are marked with grey dots for each spectrum to improve visibility.
